# A Universal Calibration Device for an Air Flow Sensor of the VAV Terminal Unit

**DOI:** 10.3390/s22155797

**Published:** 2022-08-03

**Authors:** Heng Zhang, Hui Cai, Xin Zhang, Wenjian Cai, Zhaoqin Yin

**Affiliations:** 1 College of Mechanical and Electrical Engineering, China Jiliang University, Hangzhou 310018, China; 1601400413@cjlu.edu.cn; 2 College of Electrical Engineering, Zhejiang University, Hangzhou 310027, China; zhangxin_ieee@zju.edu.cn; 3 EXQUISITUS, Centre for E-City, School of Electrical and Electronic Engineering, Nanyang Technological University, Nanyang Avenue, Singapore 639798, Singapore; ewjcai@ntu.edu.sg; 4 College of Metrology and Measurement Engineering, China Jiliang University, Hangzhou 310018, China; yinzq@cjlu.edu.cn

**Keywords:** air flow, calibration, VAV terminal units, HVAC, sensor

## Abstract

In this paper, a new calibration device for an air flow sensor of the VAV terminal unit is designed. Multi-aperture air outlets are designed to meet the calibration requirements of the air flow sensor in a variety of measurement range. The device can calibrate the air flow sensors of different types of VAV terminal unit by a movable flow rectifier without repeating the design of a different calibration pipeline. The Raspberry PI is used to design the high-performance GUI interface and controlling algorithm to achieve a one-button intelligent calibration. The air flow sensors in three different types of VAV terminal units are used to calibrate the experiment. After testing, the differential pressure value measured by the air flow sensor can accurately measure the air flow within the accuracy of 5% after the formula conversion. The conversion from differential pressure values to air flow values requires precise calibration in order to establish an accurate air flow equation, and here the calibration device plays a key role. The negative effect caused by the distance between the flow rectifiers and the VAV terminal unit is discovered. In other words, the distance between the inlet flow rectifier and the air inlet of VAV terminal unit should be kept as close as possible, or within a range of 2~3 cm. Moreover, the distance between the air outlet of VAV terminal unit and the middle-flow rectifier should be kept as close as possible; otherwise, any slight gap will cause a huge error in the calibration result. The research contributes to the further study of airflow sensing technology through the conversion and calibration of differential pressure measurements to accurate air flow values.

## 1. Introduction

In many countries, modern buildings account for 30% or more of the total energy consumption [1]. Furthermore, in modern buildings, the energy consumption from modern heating, ventilation, and air conditioning (HVAC) systems accounts for a large part of the total energy consumption [2,3]. Therefore, controlling the energy consumption of HVAC systems has great strategic significance in the green economy [4,5]. As one of the core systems of HVAC, variable air volume (VAV) systems are important to improve indoor air quality and supply heating and cooling for buildings [6,7]. The VAV terminal unit also plays a major part in the energy consumption of HVAC systems. Research on the energy consumption control of the VAV terminal unit has positive significance for the energy consumption control of the HVAC system [8,9].

VAV terminal units connect temperature and the humidity sensor, the CO_2_ sensor, and the air flow sensor [10,11]. These sensors monitor the operational status of HAVC. The air flow sensor measures the differential pressure of the VAV terminal units and the data are sent to the VAV terminal units as a signal [12]. The VAV terminal unit converts the data of differential pressures into air flow value by a specific formula and compares the air flow value with the set point which is determined based on heating or cooling or ventilation requirements [13]. If the air flow value measured by the air flow sensor is significantly different from the set point, the VAV terminal unit controls the actuator to open the damper or close it [14]. Moreover, the air flow measurements of air flow sensors also decide the power of the fan which controls the air flow value to the required value [15,16]. Obviously, the accuracy of air flow sensors is critical to the accuracy of airflow calculations for HVAC systems. However, because of the errors during the installation and unsuspected environmental factors, the accuracy of sensors is always unsatisfactory [17,18,19]. Errors will negatively affect the HVAC system. If the air flow measurement of the air flow sensor is lower than the actual air flow value, HVAC systems will consume more energy. If the air flow measurement of the air flow sensor is higher than the actual air flow value, it will not be able to meet the ventilation requirements of the building [20,21,22]. 

In the production practice of VAV terminal units, the accuracy of the air flow measurement needs to be evaluated and calibrated, so as to ensure that it can work properly in the HVAC system and can act as an accurate terminal with a feedback environmental parameter [23]. Currently, the common calibration scheme states that the producer adopts a set of calibration equipment whose caliber corresponds with the caliber of the VAV terminal units [24]. However, VAV terminal units are very different in size and caliber under different circumstances. Therefore, when producers choose this method to calibrate the accuracy of air flow measurement, they need to design calibration equipment with corresponding specifications for each type and caliber of VAV terminal units. This method increases the production costs of VAV terminal units, as well as the difficulty and complexity of calibration. The calibration devices of a different air flow sensor model of the VAV terminal unit are quite different, and a large amount of wealthy and human resources are needed for structural modeling and design in production. At the same time, it also brings a lot of waste materials. These imperfections are not conducive to the long-term development of the company.

In order to solve the above problems, a new calibration device for the air flow sensor of the VAV terminal unit is designed in this paper. At the same time, the control interface and intelligent calibration algorithm are also designed to achieve one-touch intelligent calibration. Firstly, this paper introduces the structure of the calibration device, the control interface, and the calibration process. Secondly, the corresponding conversion equation is obtained by fitting the differential pressure data and the standard air flow data. Finally, it is demonstrated experimentally that the calibration device can achieve good calibration results for the air flow sensor of different models of VAV terminal units. The influence of the position of the VAV terminal unit and rectifiers on the calibration accuracy is also analyzed experimentally.

## 2. System Scheme

### 2.1. Device Scheme

In order to simplify the calibration process of the air flow sensor of VAV terminal units, and improve the applicability of the device and reduce the cost of calibration, in this paper, a new air flow calibration system for the air flow sensor of the VAV terminal unit is designed. The device consists of five parts: an air outlet and air cavity, an electrical cabinet, a master controller, a fan, and a standard flow meter.

**Figure 1:**
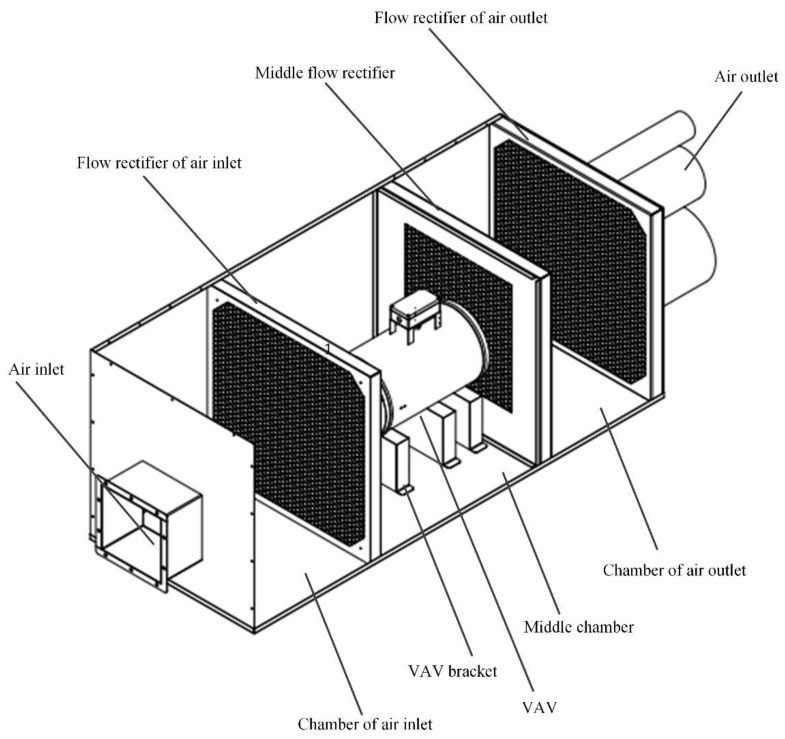
Internal structure of the device.

The internal structure of the air cavity is shown in Figure 1. It is divided into three flow rectifiers to three chambers from front to back. Based on this, the flow rate will become more stable after going through three flow rectifiers. This is very effective for improving the accuracy of calibration. The two chambers on the side of the air inlet are movable, while the one on the side of the air outlet is fixed. The size of the chambers is controlled by the flow rectifier which is moved by a sliding rail. According to the specific size of the VAV terminal unit, the user should move the flow rectifier to control the size of the chamber. In this way, the device can be adapted to the air flow sensor of various VAV terminal units and become more versatile.

**Figure 2:**
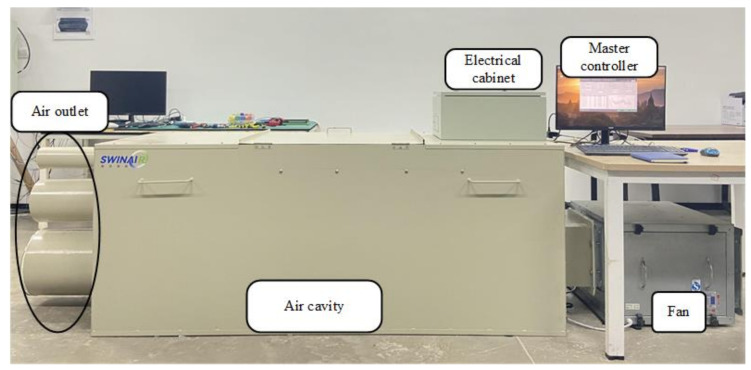
Photo of the calibration device prototype.

The photo of calibration device prototype is shown in Figure 2. The device has three air outlets of different diameters. Users can choose a suitable diameter of air outlet according to the range of air flow measurement, and the other two will be blocked by physical blocking. The electrical cabinet contains electrical switches, a switching power supply, an intermediate controller, a 485 hub, and a frequency converter. The master controller consists of a 485 adapter, a Raspberry PI, and accessories. The Raspberry PI communicates with the controller and the VAV terminal units based on Modbus. The fan is controlled by a frequency converter. The Raspberry PI sends instructions to the intermediate controller by Modbus. The intermediate controller handles the command, and outputs a 0~10 V voltage signal to control the converter. The different frequency of the converter controls the amount of air flow inside the device.

**Figure 3:**
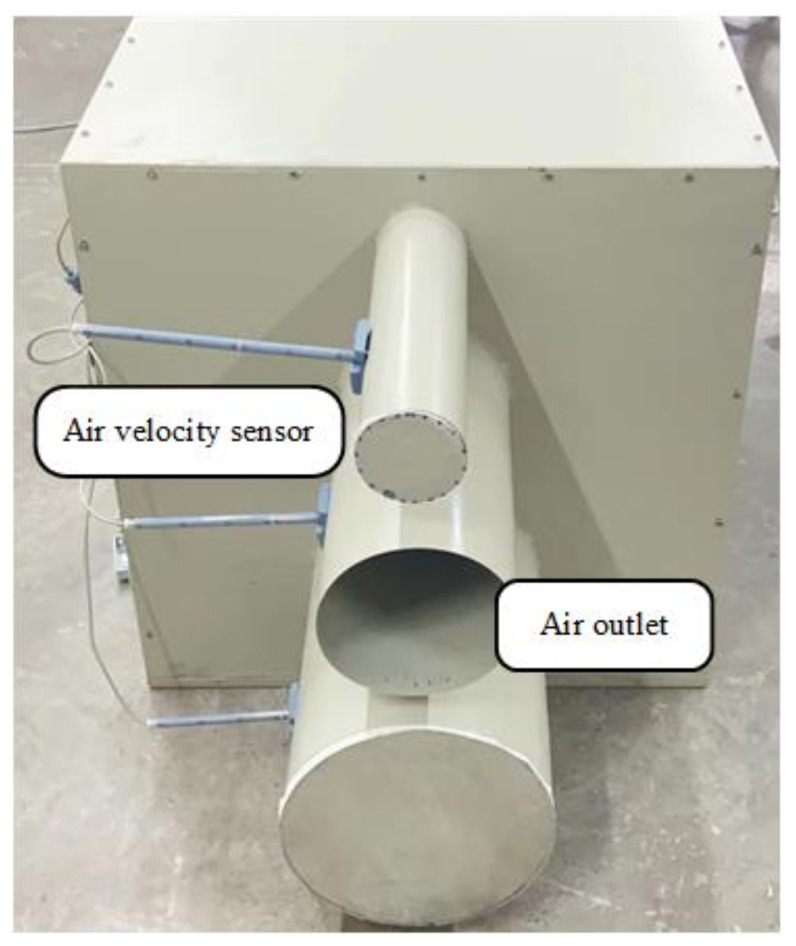
Installation of the air velocity sensor.

The Siemens air velocity sensor QVM62.1 is used to monitor the flow in air ducts and the installation, as shown in Figure 3. By converting the wind speed into a current of 4~20 mA, the intermediate controller can obtain a specific wind speed through AD conversion. Then, the standard flow value can be obtained through a specific calculation formula.

The VAV terminal unit with the air flow sensor is placed in the middle chamber. The top of the air valve bracket is flat, and it has a groove in the middle so that both square VAV terminal units and circular VAV terminal units can be placed directly on top of the bracket for calibration. In this way, the different types of VAV terminal units can be firmly placed and it can be ensured that the position of the VAV terminal units remains stable during calibration. When calibration is carried out, one side of the VAV terminal unit is close to the flow rectifier of air inlet, and the other side is close to the middle-flow rectifier. The acrylic plate placed near the middle-flow rectifier has a hole depending on the diameter of the VAV terminal units and the position of the outlet. This way, it can ensure that the air flow going through the air outlet is all from the duct of VAV terminal units.

When the user needs to calibrate the air flow sensor of different types and specifications of VAV terminal units, they only need to change the acrylic plate to control the size of the middle chamber and the air outlet. The device makes the calibration process more efficient and convenient. This new airflow calibration device reduces the amount of wasted time and resources in the calibration process for companies.

### 2.2. System Control Interface

**Figure 4:**
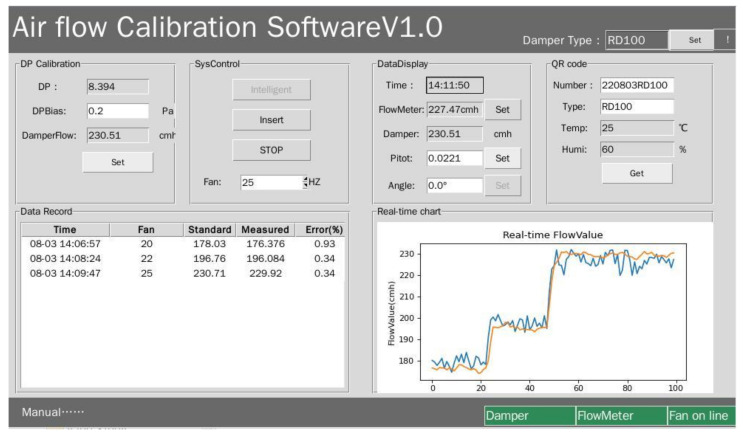
Intelligent control interface.

In order to improve the intelligence and automation of the calibration process, the GUI interface was designed by Python, as shown in Figure 4. The GUI interface runs on Raspberry PI’s Linux system. The Raspberry PI communicates with the intermediate controller and the VAV terminal unit based on Modbus. The VAV terminal unit transmits various environmental parameters and system parameters to the Raspberry PI by Modbus.

This interface provides two calibration modes: one-click intelligent calibration and manual calibration, which can adapt to a variety of environments and requirements. The relevant data in the calibration process are recorded in real time, and the calibration records are displayed in table form, making it convenient for the user to master the calibration process of the monitoring system. In order to ensure the long-term validity and traceability of calibration results, all data in any calibration process are recorded in the SQLite database.

Before calibration, the user can click the “set” button close to the label of “Damper Type” to choose the type of VAV terminal unit. The selected parameter is used to find its corresponding calibration range and the size in the database so that the calibration range can be intelligently set to improve the efficiency of calibration.

In addition, for the air flow sensor of the same type of VAV terminal unit, the characteristics of the system are relatively consistent, and the current intelligent calibration parameters can be saved by clicking OK in the dialog box which pops up after calibration, so that if the user needs to calibrate the air flow sensor of the same type VAV terminal units in the future, the historical parameters can be directly used for rapid calibration. Therefore, the work will be more efficient.

### 2.3. Calibration Process

**Figure 5:**
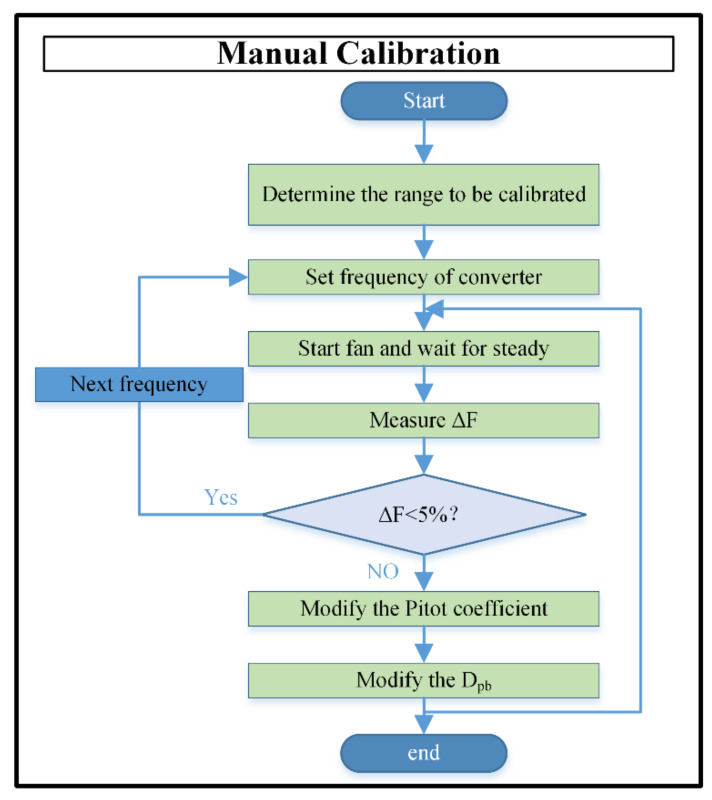
Flowchart of manual calibration.

The main calibration process is divided into two parts: hardware preparation and software operation. A flowchart of the manual calibration is shown in Figure 5.

For the hardware preparation part, users should determine the model and the air flow measurement range of the air flow sensor before each calibration, and choose the appropriate outlet caliber according to the maximum and minimum air flow. Then, users need to choose a suitable acrylic plate according to the VAV terminal unit caliber and place the VAV terminal units on the stand.

If the user chooses the manual mode, the calibration range should be determined, and the frequency of the frequency converter should be set at certain intervals according to the range. The user manually sets the frequency of the frequency converter to control the power of the fan and then waits for the stable operation of the system, before changing the value of $D_{pb}$ and the Pitot according to the error rate between the air flow measurement measured by the air flow sensor of the VAV terminal unit and that measured by a standard flowmeter. The user selects different a converter frequency according to the measurement range and repeats the above steps.

**Figure 6:**
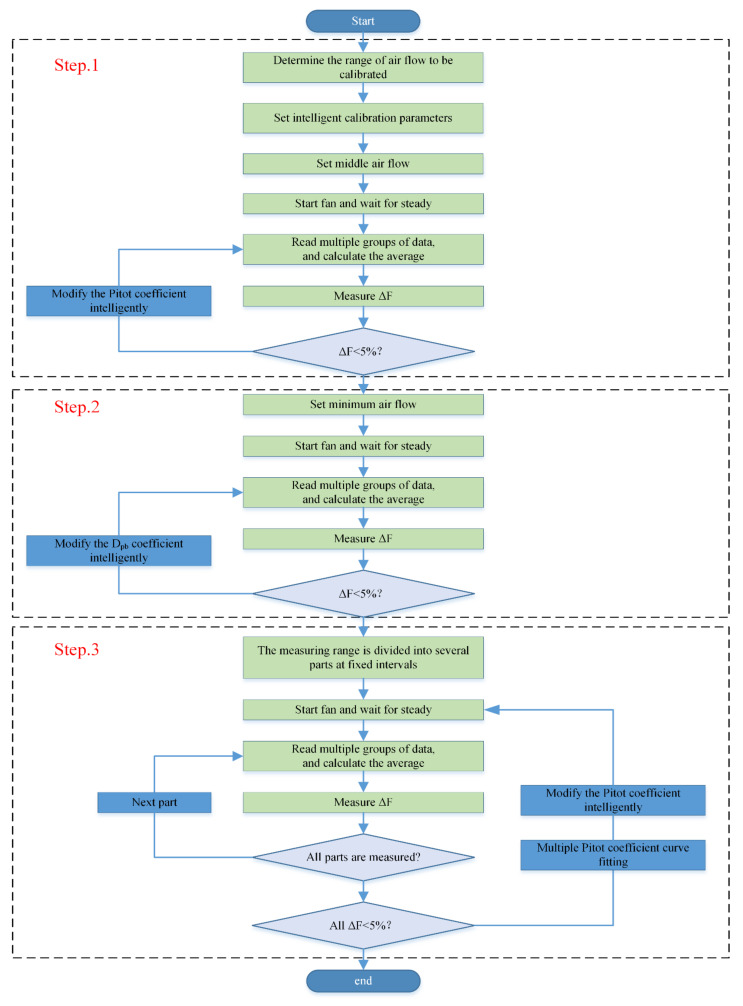
Flowchart of one-click intelligent calibration.

The whole intelligent calibration process is divided into three steps. The flowchart is shown in Figure 6.

In step 1, the frequency of the converter is firstly set to half of the air flow measurement range, so as to quickly obtain a base Pitot coefficient The system starts the fan and waits for the system to stabilize. When the data are stable, the system reads multiple groups of data and calculates the average. There is an error rate Δ*F* between the standard air flow measured by the flow meter and the air flow measured by the air flow sensor of the VAV terminal unit, and the relationship is as follows:

(1) $$\Delta F=\frac{Q_{F}-Q_{D}}{Q_{F}}\cdot 100\%$$

where Δ*F* is the error rate of the standard air flow measured by the flow meter and the air flow measured by the air flow sensor of VAV terminal units, $Q_{F}$ is the air flow measured by the standard flow meter, and $Q_{D}$ is the air flow measured by the air flow sensor of VAV terminal units.

Equation (1) is used to obtain the value of ΔF after calculating the average value of standard flow meter and the average value of VAV terminal units. Then, the system judges whether the error requirement of 5% is met. If not, the value of the Pitot tube coefficient will be updated after calculations, and the preceding operations are repeated.

In step 2, the $D_{pb}$ is the pressure measurement deviation and is determined by the calibration of a low air flow value. Firstly, the system controls the fan to reach the minimum air flow in the range, starts the fan, and then waits for a period of time until the air flow is stable. When the data are stable, the system obtains the value of ΔF after reading multiple groups of data and calculating the average. If the 5% requirement is not met, the value of $D_{pb}$ will be updated after calculating, and the preceding operations are repeated.

In step 3, the calibration process is divided into several specific points according to the range, and the ΔF of each point is measured respectively. Thus, the error of the Pitot tube coefficient of each point is calculated. A final Pitot tube coefficient will be obtained by curve-fitting the errors of the Pitot tube coefficients corresponding to all points. Then, the steps are repeated until all points are within 5%.

At this point, the whole intelligent calibration process is completed.

## 3. Experiment and Result

### 3.1. Experiment Preparation

**Figure 7:**
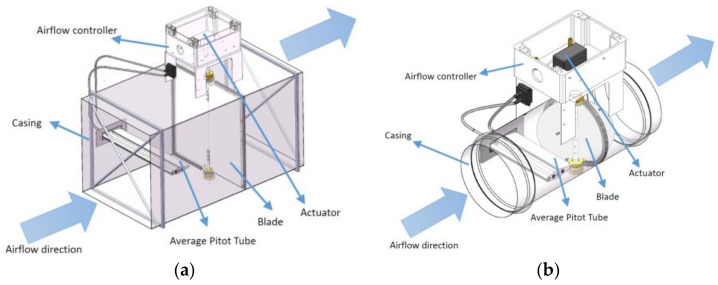
Structure of VAV terminal units. (**a**) Square VAV terminal unit; (**b**) circular VAV terminal unit.

The VAV terminal unit is an important part of the HVAC system which can be used to supply, exhaust, or mix air. No matter what changes happen in room conditions or pipe pressure, it can maintain the air flow in a required level using precise measurements and control. Measurements are always based on the differential pressure generated by the average Pitot tube (APT) and the differential pressure transducer with high precision. The two types of VAV terminal units used in this paper are shown in Figure 7. Air flow measurements are processed by the flow controller, which issues commands to the actuator and keeps the flow at a predetermined set point. Due to the difference in manufacturing, even if the same type of VAV terminal units uses the same differential pressure sensor, the measurements are different. So, the air flow sensor of each VAV terminal unit needs to be calibrated.

**Figure 8:**
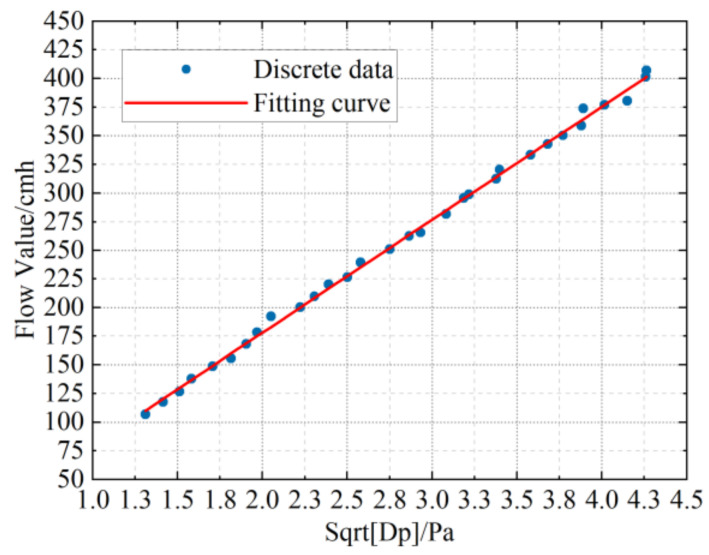
Diagram of air valve pressure difference and air flow.

The SQ150 VAV terminal unit, which is square, is used in the calibration experiment. The frequency of the fan is set according to the flow measurement range of different models. Therefore, when the frequency converter steps from 14 Hz to 44 Hz at an interval of 1 Hz, the differential pressure measured by the air flow sensor of the VAV terminal units and the outlet air flow measured by the standard flow meter at each point are recorded. The square root of every pressure difference ($D_{p}$) is calculated. Then, all the recorded discrete data are fitted with linear curves. As shown in Figure 8, all the discrete points are fitted into a straight line. It is obvious from the figure that all the points are densely distributed around the straight line. Therefore, the square root of the pressure difference measured by the VAV terminal unit shows a roughly linear trend with the actual air flow. The final fitting result of these discrete data is as follows:

(2) $$Q_{D}=98.6648\cdot \sqrt{D_{p}}-19.2505$$

where $D_{p}$ is the pressure difference value measured by VAV terminal units.

Meanwhile, according to the Bernoulli’s equation, the air flow speed is influenced by the Pitot tube coefficient, the dynamic pressure, and the fluid density:

(3) $$V=K\cdot \sqrt{\frac{2P}{\rho }}$$

where *V* is the air flow speed, *K* is the Pitot tube coefficient, *P* is the dynamic pressure measured by Pitot tube, and ρ is the fluid density.

The calculation formula of air volume is as follows:

(4) $$Q_{D}=3600\cdot V\cdot F$$

where *F* is the pipeline cross-sectional area.

Based on the above analysis, $D_{pb}$ is introduced into the relationship between pressure difference and air flow, and the expression of the relationship between pressure difference and wind flow is established as follows:

(5) $$Q_{D}=3600\cdot K_{p}\cdot \sqrt{D_{p}-D_{pb}}$$

where $K_{p}$ is the Pitot coefficient, and $D_{p}$ is the pressure difference measured by the air flow sensor of VAV terminal units.

### 3.2. Measurement Range

The measurement range of the flow sensors is mainly limited by the aperture of air outlet. In this device, there are three different apertures of air outlets. The formula of the standard flowmeter is shown as follows:

(6) $$Q_{F}=3600\cdot V_{F}\cdot S_{o}$$

where $V_{F}$ is the wind speed and $S_{o}$ is the cross-sectional area of the air outlet.

The cross-sectional area of the biggest one in three air outlets is $0.0225\pi \;m^{2}$, and the maximum wind speed that the fan can blow is 15 m/s. Therefore, the maximum range of air flows is 0 to 3815.1 cmh. The range of air flow measurement corresponding to three air outlets is shown in Table 1. In addition, the volume size of the VAV terminal unit is limited due to the limited space in the chamber. The maximum length of the VAV terminal unit that can be measured is 85 cm. If the VAV terminal unit is square, the maximum cross-sectional area is 2500 cm^2^. If the VAV terminal unit is round, the maximum cross-sectional area is $625\pi \;{\mathrm{cm}}^{2}$. If the VAV terminal unit meets the above range requirements, then calibration tasks can be performed with this device.

The design of the groove structure can stabilize the round and square VAV terminal unit to prevent the interference caused by jitter in the process of calibration. Therefore, the device does not make special requirements for the shape of the VAV terminal unit, whether the square or round VAV terminal unit can be placed in the device steadily, and other common shapes can also be adapted. The equipment can meet the calibration requirements of the air flow sensor of most types of VAV systems in the market.

### 3.3. Calibration Process

**Figure 9:**
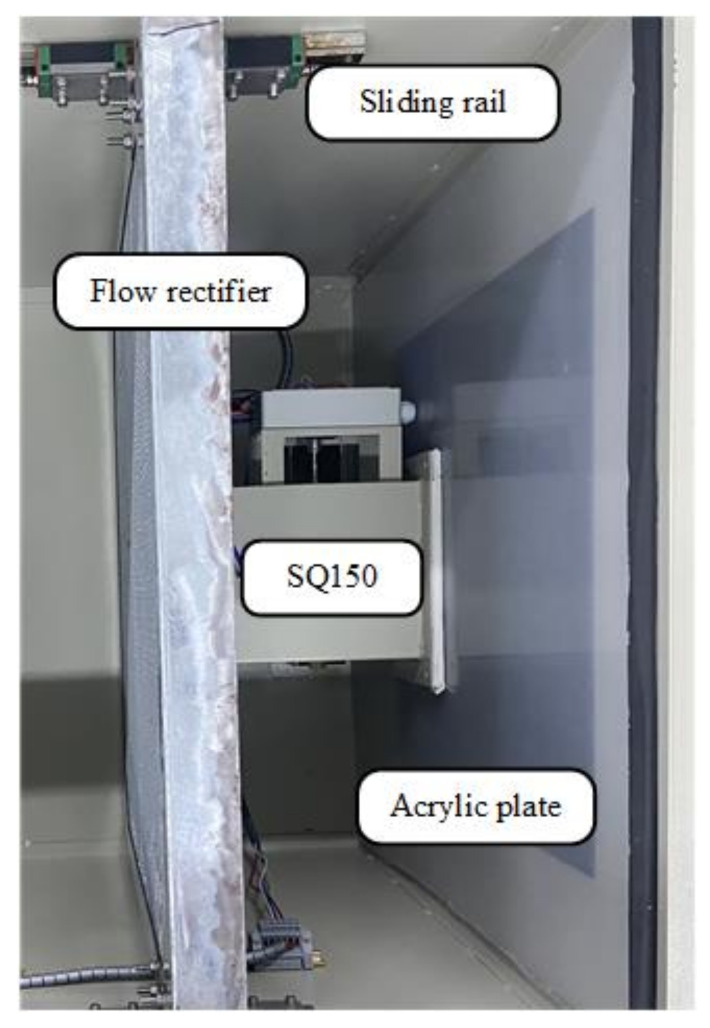
SQ150 hardware preparation.

Hardware preparation is required before calibrating the air flow sensor of the VAV terminal unit. As shown in Figure 9, a SQ150 VAV terminal unit is ready for calibration. The SQ150 is placed on the bracket and the acrylic plate is close to the air outlet of VAV terminal unit and flow rectifier by cutting a suitable caliber. In addition, it is convenient to move the flow rectifier on the slide so that it can be close to the air inlet of the VAV terminal unit.

**Figure 10:**
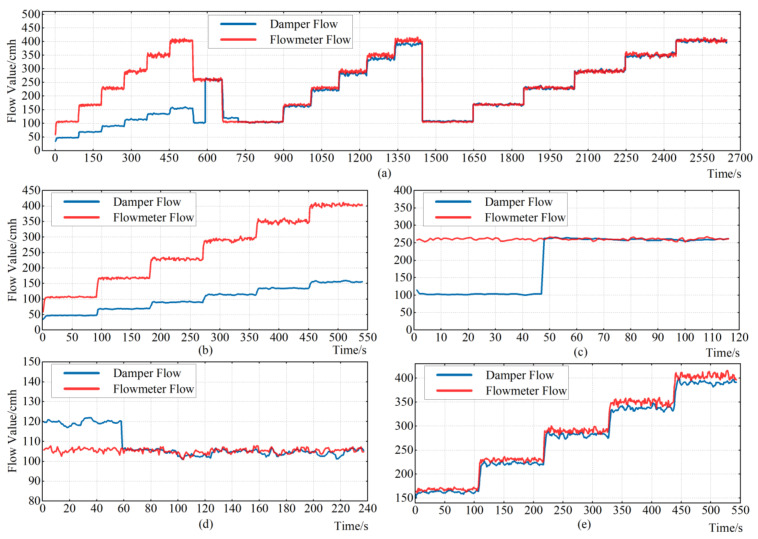
SQ150 air flow calibration process. (**a**) The comparison trend of air flow measurement measured by the air flow sensor of the VAV terminal unit and air flow measurement measured by standard flow meter; (**b**) the trend between the air flow measurement measured by the air flow sensor of the VAV terminal unit and the standard flow meter before calibration; (**c**) calibration of $K_{p}$ at the middle position of the air flow measurement range; (**d**) calibration of $D_{pb}$ in the low flow; (**e**) the trend between the air flow measurement measured by the air flow sensor of the VAV terminal unit and the standard flow meter before calibrating $K_{p}$.

During the calibration process of air flow measurement for SQ150, a comparison trend of air flow measurement measured by the air flow sensor of VAV terminal unit and air flow measurement measured by standard flow meter is shown in Figure 10a. In order to show the complete calibration process, a comparison of air flow before and after calibration is added. It can be clearly seen from the figure that the air flow measurement measured by the air flow sensor of the VAV terminal unit is adjusted from a large error at the beginning to a small error at the end.

Before calibration, the trend between the air flow measurement measured by the air flow sensor of the VAV terminal unit and the standard flow meter is shown in Figure 10b. There is an error rate Δ*F* between the air flow measurement measured by the air flow sensor of the VAV terminal unit and the air flow measurement measured by the standard flow meter, and the relation equation is shown as Equation (1).

The error rate ΔF represents the accuracy of the air flow measurement of the air flow sensor of VAV terminal units. In order to ensure that the air flow sensor of the VAV terminal unit can measure accurately and achieve high accuracy, the Pitot coefficient is constantly corrected during the calibration process so that the error rate Δ*F* is constantly reduced until it is controlled within the required error range. The difference between the Pitot coefficient and the expected Pitot coefficient ΔPitot is positively correlated with the air flow error rate Δ*F*, and the relation equation is as follows:

(7) $$\Delta K_{p}=\frac{\Delta F*P_{F}}{3600*\sqrt{D_{p}-D_{pb}}}$$

where Δ$K_{p}$ is the difference between the actual Pitot coefficient and the expected Pitot coefficient.

As shown in Figure 10c, in order to improve the speed of calibration, the Pitot coefficient is rapidly calibrated at the middle position of the air flow measurement range. In this process, the Pitot coefficient is constantly updated based on Equation (7) until the Δ*F* within the error range. The actual Pitot coefficient is as follows:

(8) $$K_{p}=\Delta K_{p}+K_{pold}$$

where $K_{pold}$ is the old Pitot coefficient.

The pressure difference measured by the air flow sensor of the VAV terminal unit is alike at each air flow value. The Pitot and $D_{pb}$ mainly affect the measurement accuracy. Due to the measurement characteristics, the air flow sensor is more sensitive to $D_{pb}$ when the flow set point is low. Meanwhile, it can be seen from Equation (5) that the air flow and $D_{p}$ show a positive correlation trend. $D_{pb}$ directly acts on $D_{p}$, so the lowest value of air flow range is used for the calibration of $D_{pb}$. The relation between Δ$D_{pb}$ and air flow is as follows:

(9) $$D_{pb}\;=-\frac{{Q_{F}}^{2}-{Q_{D}}^{2}}{3600^{2}*{K_{p}}^{2}}$$

As shown in Figure 10d, after rapid Pitot calibration at the mid-range position, there is still a large error at the low-flow position. After the calibration of $D_{pb}$ with Equation (9), the error in the low-flow position is eliminated and the accuracy is greatly improved.

In order to ensure that all values within the measurement range can meet the measurement error requirements, the calibration process is divided into multiple points, as shown in Figure 10e. Because of the influence of complex environmental factors, each point has a special deviation, and the expected Pitot tube coefficient between each point has a complex coupling relationship. In order to achieve high accuracy within the measurement range, the linear fitting of $\Delta K_{p}$ measured at each point is carried out.

In order to further reduce the error of measurement, Pitot coefficient fitting calibration is carried out to decouple the coupling relationship of the Pitot coefficient at each point.

**Figure 11:**
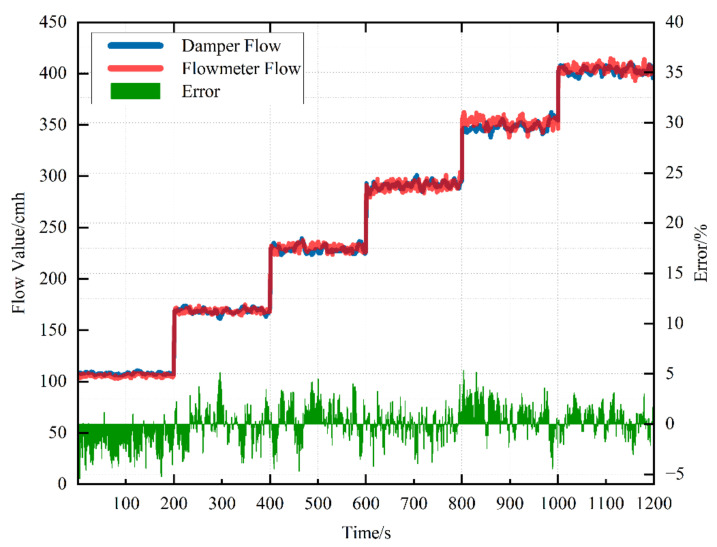
Data comparison diagram of SQ150 after stabilization.

After SQ150’s calibration, the $K_{p}$ and $D_{pb}$ are set. In order to further verify the correctness of the calibration results, the $K_{p}$ and $D_{pb}$ are not adjusted and the measurement is compared again. A comparison diagram of the air flow measured by the air flow sensor of VAV terminal units and the air flow measured by the standard flow meter and its error trend are shown in Figure 11. It is obvious that the air flow measured by the air flow sensor of VAV terminal units and the air flow measured by the standard flow meter almost coincide after calibration. Although there are slight fluctuations caused by environmental and measurement disturbances, the overall trend remains within 5% of the error requirement.

**Figure 12:**
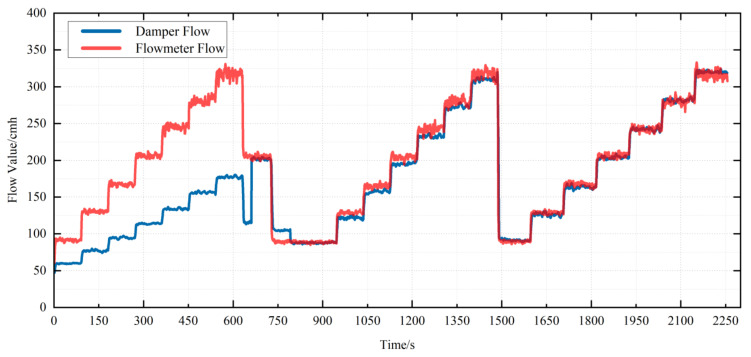
Air flow calibration process of RD150.

**Figure 13:**
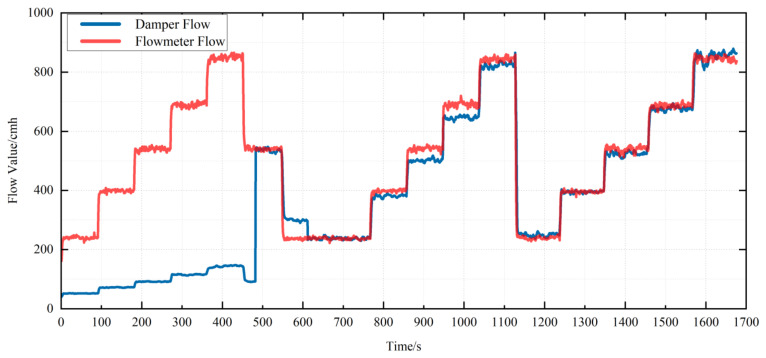
Air flow calibration process of RD250.

**Figure 14:**
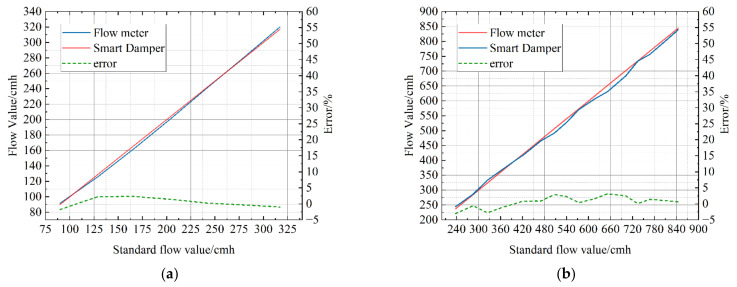
Data comparison diagrams of RD150 and RD250 after stabilization. (**a**) The errors of RD150 at the end of calibration; (**b**) the errors of RD250 at the end of calibration.

In order to verify the applicability of the system to the air flow sensor of different types of VAV terminal units, RD150 and RD250, which are circular, are selected for calibration experiments. The data in the calibration process are shown in Figure 12 and Figure 13. It can be seen from the data in the figure that the measurement accuracy of the air flow sensor of VAV terminal units is significantly improved after calibration. The errors of RD150 at the end of calibration are shown in Figure 14a and the errors of RD250 at the end of calibration are shown in Figure 14b. Obviously, the overall trend of errors remains within 5%.

Through the above calibration tests on the air flow sensors of square and circular VAV terminal units, we found that the device can be well adapted to the calibration of air flow sensors of different types and different tube diameters of VAV terminal units, and the errors can be all controlled within 5%. Thus, the system works equally well with square and circular geometries.

### 3.4. Influence of Distance between Flow Rectifier and Air Inlet of VAV Terminal Unit

The flow rectifier is a device which can change an irregular flow of air into a regular flow or a rotating flow into a straight flow. In this way, all of air flow that enters the VAV terminal units will firstly go through the flow rectifier and the negative effects of the irregular air flow will be avoided. Moreover, adopting the flow rectifier can simulate the actual pipeline environment and ensure the accuracy of calibration.

The flow rectifier greatly affects the calibration process as an important part of the system. The distance between the flow rectifier and the air inlet of the VAV terminal unit greatly influences the calibration. Therefore, in this test, we control the distance to 0 cm and calibrate the VAV terminal unit to obtain some useful parameters. The tests of distance from 0 cm to 7 cm are based on those parameters.

**Figure 15:**
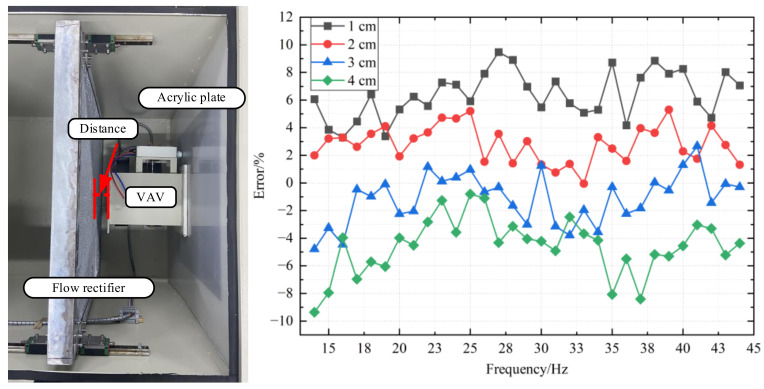
Influence of different distance on measuring accuracy.

The error between the air flow measured by the air flow sensor of the VAV terminal unit and the air flow measured by the standard flow meter is recorded at different distances by constantly adjusting the distance. Meanwhile, the major errors of different distance are shown in Figure 15. It is obvious that the error is perfectly maintained within 5% when the distance is 0 cm. When the distance increases slowly, there is a slight oscillation from 0 to 1 cm, and the error expands to 10%. In the range of 2~3 cm, the error recovers within 5%. When the distance exceeds 3 cm, the error continues to widen and begins to produce violent oscillations. In addition, when the distance is in range of 2~3 cm, although the error is still within 5%, it is obviously oscillating. When the distance becomes larger, the effect of flow rectifiers on the air flow gradually weakens, resulting in the air flow becoming irregular and the accuracy of measurement being affected.

According to the results of the experiment, the distance between the flow rectifier and the air inlet of the VAV terminal unit greatly influences the results of calibration. In order to achieve a good effect of calibration, the distance is expected to reach 0 cm, but the distance within 2~3 cm is also feasible. Therefore, in the process of calibration, the VAV terminal unit does not need to be close to the rectifier to achieve the desired calibration accuracy. Therefore, in the ordinary use of the process, the placement requirements are much lower.

### 3.5. Influence of Distance between Middle-Flow Rectifier and Air Outlet of VAV Terminal Unit

The distance between the air outlet of VAV terminal unit and the flow rectifier also affects the accuracy of calibration. To ensure that the airflow into the third chamber comes only from the inlet or duct of VAV terminal unit, an acrylic plate is placed between the VAV unit and the middle-flow rectifier to cut a hole according to the diameter of the VAV terminal unit and the position of the outlet. When the distance between the air outlet of VAV terminal unit and the flow rectifier exists, a large amount of air flow directly goes through the flow rectifier without going through the duct of VAV terminal units.

**Figure 16:**
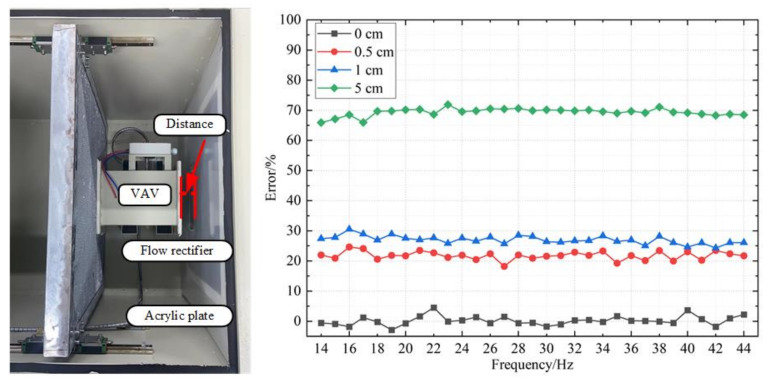
Influence of different distances on the measuring accuracy.

Based on this, VAV terminal units are placed in different positions to make the distance between the air outlet of VAV terminal unit and the flow rectifier exist for the experiment. In this test, we control the distance to 0 cm and calibrate the VAV terminal unit to obtain some useful parameters. The tests of distance from 0 cm to 5 cm are based on those parameters. The error between the air flow measured by the air flow sensor of VAV terminal units and the air flow measured by the standard flow meter is recorded at different distances by constantly adjusting the distance, as shown in Figure 16. From the figure, we can discover that when the distance is as low as possible, the corresponding errors are within 5%. However, when we increase the distance, although the distance is relatively small, the errors will change dramatically. Moreover, as the distance increases, the error will continue to increase.

Therefore, during the calibration process, the air outlet of VAV terminal unit should be placed closely with the flow rectifier to ensure that all the air flow into the outlet can pass through the duct of the VAV terminal unit and ensure the accuracy of measurement results. The air outlet and the middle-flow rectifier need to be tightly fitted, so it is recommended to add sealing measures for better calibration effect.

### 3.6. Influence of Placing Angle of the VAV Terminal Unit

**Figure 17:**
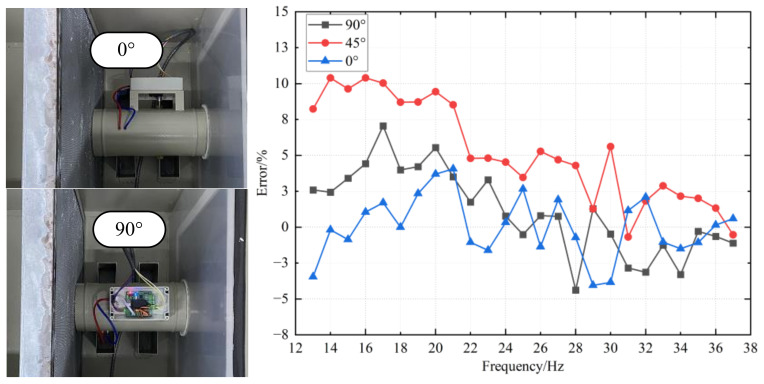
Placing angle of different distance on measuring accuracy.

In the calibration experiments, when the VAV terminal unit is placed on the bracket, the different angles of VAV terminal unit will also influence the calibration result. This is caused by the measurement characteristics of the air flow sensor. Therefore, we calibrate the VAV terminal unit at 0° and recode its system parameters. With these parameters, experiment data of 0°, 45°, and 90° are recorded, respectively. By analyzing the errors between the data measured by the VAV terminal unit and the data measured by the standard flow meter at different angles, a diagram of errors is shown in Figure 17. It is evident that the error of VAV terminal unit is calibrated within 5% at 0°, while there is a slight oscillation at 90°, especially at low air flow values. In addition, we also carry out an experiment on the placement position of 45°. An angle of 45° will negatively influence the results, causing severe jitter of the measurement results. This is because the VAV terminal unit cannot be fixed well in 45°, and the unstable flow of air flow disturbs the VAV terminal unit. Therefore, the measurement accuracy is seriously affected.

## 4. Conclusions

In this paper, we propose a new air flow calibration device for the air flow sensor of VAV terminal unit. The device integrates all the equipment needed in the calibration process. We design three different specifications of the outlet using standard flow meters to measure the air flow of the outlet. Therefore, we can expand the calibration range of the device. At the same time, the main chamber is divided into three parts by three flow rectifiers. The two chambers close to the fan are movable, which are realized by sliding the guide rails. When VAV terminal units of different lengths need to be placed on the bracket, the size of the middle chamber can be adjusted by controlling the position of the flow rectifier via the slide rail.

Python, a high-performance scripting language, is used to design the upper computer software, and the system parameters are displayed in real time. We design the easy operation method to reduce the difficulty of calibration and the labor cost of the manufacturer. In addition, two modes of one-key intelligent calibration and manual calibration were designed to make the device suitable for different calibration occasions.

Through the calibration experiments on the air flow sensor of three different types of VAV terminal units, we prove the feasibility of the calibration device. The accuracy of all the air flow sensor of the calibrated VAV terminals is within 5%. At the same time, the influence of the distance between the flow rectifier and the VAV terminal unit is analyzed experimentally. The distance between the flow rectifier and the inlet of VAV terminal unit should be as close as possible, or 2~3 cm is also appropriate. The distance between the middle-flow rectifier and the VAV terminal unit outlet should be as close as possible, as any deviation of distance may lead to huge deviation in the measurement results. In addition, the influence of the angle of VAV terminal unit on calibration results is presented through experiments. The accuracy of calibration can only be ensured when the VAV terminal unit is placed horizontally. Any other angles can lead to calibration errors.

Therefore, the device can be applied to the air flow sensor of many types of VAV terminal units. When different VAV terminal units are calibrated, only the acrylic plate and air outlet need to be replaced. There is no need to design a separate calibration system for each VAV terminal unit, like with the traditional calibration method. This integrated design can greatly improve the economic benefits of enterprises. Furthermore, the device can be helpful for enterprises to produce a VAV terminal unit with higher accuracy and has good practical application value.

The calibration of the air flow sensor of the VAV terminal unit is a key to VAV terminal units’ proper use and a technically difficult task. The air flow sensor is used in the VAV terminal unit to measure the air flow rate, but it can only directly measure the differential pressure. The conversion from differential pressure values to air flow values requires precise calibration in order to establish an accurate air flow equation, and here the calibration device plays a key role. The research contributes to the further study of airflow sensing technology through the conversion and calibration of differential pressure measurements to obtain accurate air flow values.

## Figures and Tables

**Table 1:** Range of air flow measurements corresponding to three air outlets.

Cross-Sectional Area $\left(m^{2}\right)$	Range of $V_{F}\;(m/s)$	Range of $Q_{F}\;(\mathrm{cmh})$
0.0025π	0~15	0~423.90
0.01π	0~15	0~1695.6
0.0225π	0~15	0~3815.1

## Data Availability

Not applicable.
